# Molecular diameters of rarefied gases

**DOI:** 10.1038/s41598-022-05871-y

**Published:** 2022-02-08

**Authors:** S. Kunze, R. Groll, B. Besser, J. Thöming

**Affiliations:** 1 Chemical Process Engineering, Universität Bremen, Leobener Str. 6, 28359 Bremen, Germany; 2 Center of Applied Space Technology and Microgravity, Universität Bremen, Am Fallturm 2, 28359 Bremen, Germany

**Keywords:** Engineering, Atomic and molecular physics, Fluid dynamics

## Abstract

Molecular diameters are an important property of gases for numerous scientific and technical disciplines. Different measurement techniques for these diameters exist, each delivering a characteristic value. Their reliability in describing the flow of rarefied gases, however, has not yet been discussed, especially the case for the transitional range between continuum and ballistic flow. Here, we present a method to describe gas flows in straight channels with arbitrary cross sections for the whole Knudsen range by using a superposition model based on molecular diameters. This model allows us to determine a transition diameter from flow measurement data that paves the way for generalized calculations of gas behaviour under rarefied conditions linking continuum and free molecular regime.

## Introduction

In many natural processes and technical applications, the size of a gas molecule is a key property that influences the rate at which lungs can absorb oxygen^1^, affects chemical reactions^2^, defines the drag on wind turbines^3^ and limits diffusion across membranes^4^, mainly because the size strongly affects the mobility of a molecule. For unification, the size is often generalized as the diameter of an equivalent sphere. However, a molecule is neither a rigid object nor has, in most cases, a spherical shape. Its volume is made up of electron orbitals representing the probability of finding an electron at a specific position. It is, therefore, impossible to state one definite diameter of a molecule. As a result, different operationally defined diameters are used for various scenarios.

The van der Waals diameter is a prominent measure and is typically used to calculate the properties of condensed matter^5^. The van der Waals diameter is obtained by crystallographic experiments such as X-ray diffraction, zero point density data^5^ or the molar volume of solids^6^. This diameter is also applied in the van der Waals equation of state to account for the nonideal behaviour of gases under high pressure^7,8^.

Transport processes such as the absorption of oxygen in the lungs or gas transport through a membrane are strongly influenced by diffusion effects. The diffusion coefficients of molecules can be estimated based on their diameter. Here, the kinetic diameter of a molecule is frequently used, which is obtained from molecular sieving experiments^4^.

The drag on wind turbines is mainly a result of gas viscosity. Viscosity is also an effect resulting from the molecular diameter of a gas. Measured viscosity, usually obtained at normal pressure, can therefore be used to estimate molecular diameters. These diameters are typically utilized to calculate the mean free path λ^9^, which plays a crucial role in gas kinetics:

1 $$\lambda =\frac{{k}_{B}T}{\sqrt{2}\pi {d}^{2}p}$$

The kinetic theory of gases can be utilized to describe the behaviour of a gas^10^. The movement of a gas molecule is strongly affected by its interaction with its environment involving collisions with other gas molecules or the surrounding geometry. The frequency of intermolecular collisions is determined by the mean free path of a molecule. The lower the pressure is, the less likely a collision is between two molecules within a given time. In contrast, the smaller the surrounding geometry is, the more likely a collision is between gas molecules and the surface. The ratio between the mean free path and the characteristic length of the geometry in question is represented by the dimensionless Knudsen number *Kn*^11^.

Under elevated pressure conditions or in larger geometries, the Knudsen number is close to zero. This so-called continuum regime is present in many technical applications. Examples are everyday phenomena under standard conditions, such as the abovementioned viscosity effects, as well as high-pressure reaction engineering^12^. At ultrahigh vacuum or in relatively small geometries, however, the Knudsen number becomes very large. This is an important property of scanning electron microscopy in which gas molecules do not interfere with the electron beam. Another example where the Knudsen number tends to infinity is molecular sieves. Here, gas molecules diffuse through a pore structure where pore sizes are on the same order of magnitude as the molecular diameter^13^. Both extremes are well described by existing models, such as the Navier Stokes equations for continuum conditions^11^ or the Smoluchowski equation for free molecular flow^14^. Each of these models has analytical solutions for simple geometries.

**Figure 1:**
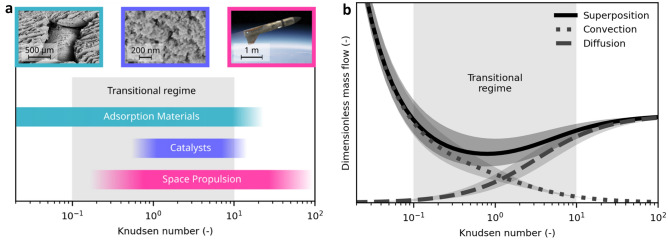
Rarefied gas flow in technical applications and its dependence on the molecular diameter. (**a**) Typical Knudsen regions of technical applications. Materials for gas adsorption (cyan) can have pore sizes ranging over multiple orders of magnitude. In combination with large changes in pressure or temperature in swing-adsorption applications, the result is a wide Knudsen range. In catalysis (blue), products and educts often face small structures at high temperatures and Knudsen numbers larger than unity at atmospheric pressure can be observed. In space applications such as propulsion modules (magenta), the Knudsen numbers tend to be very large due to the low pressure in orbit. See Supplementary Text 1 for details on the Knudsen ranges. Left image: SEM image of a hierarchical structured zeolite adsorbent. Center image: SEM image of a Sm_2_O_3_ xerogel catalyst. Right image: Depiction of the ReFEx vehicle^21^. (**b**) Schematic illustration of the individual transport terms of the superimposed model and their variation with the Knudsen number. The convective part decreases with rising Knudsen number while the diffusive part becomes dominant at Knudsen numbers larger than unity, because of the increasing mean free path. The diffusive part converges to a constant value at high Knudsen numbers where the surrounding geometry is the limiting factor for diffusion. The sum of both components reproduces the typical and well- known Knudsen minimum at approximately *Kn* = 1. The influence of the molecular diameter on the predicted mass flow is illustrated by the shaded areas.

In many applications, however, Knudsen numbers are neither close to zero nor tend to infinity. Examples for applications that are based on gas flow under these so-called rarefied conditions are swing-adsorption processes, catalysis, and space propulsion systems with typical Knudsen ranges depicted in Fig. 1a. For several modelling approaches addressing gas flow under rarefied conditions^15^ including models using superposition^16,17^, fitting coefficients are needed, which lack a clear physical meaning. For generalizability and understanding of the involved effects, distinct physical meaning is desirable. Recent works are presented by Xie et al.^18^ and Groll et al.^19^ and a comprehensive overview of macroscopic modelling techniques for rarefied gas flows is given by Struchtrup^20^.

When modelling such scenarios, one has been limited to choosing between molecular diameters obtained at either *Kn* → 0 or at *Kn* → ∞. Because the effective diameter of a molecule depends on the surrounding conditions, those diameters are most likely not sufficiently accurate for covering the full Knudsen range. A diameter relating to rarefied conditions with emphasis on the transitional regime^11^ could fill this gap.

To determine molecular diameters under rarefied gas conditions, we propose a method using measurements of rarefied gas flows in straight channels. Based on well-established models for convective, diffusive and free molecular flow, a superimposed form is introduced covering the whole Knudsen range. The resulting model is predictive and applicable to any channel cross section. We show that this analytical description can be utilized to obtain molecular diameters corresponding to rarefied conditions.

## Results

The proposed analytical description for rarefied gas flow is composed of models for convective and diffusive flow. Mass flow components and total mass flow for an exemplary case are depicted in Fig. 1b; for case details, see Supplementary Text 2. The sensitivity of the mass flows to a change in the molecular diameter of ± 25% is illustrated as an area around the curves. To visualize the whole Knudsen range and to exclude the influence of channel size, the mass flows are shown relative to a mass flow often called “Knudsen diffusion”. This is the self-diffusive flow, see Eq. (18) in the Methodology section, with a mean free path equal to the hydraulic diameter *D*_*H*_ = *4A/*Π, where *A* is the channel cross sectional area, and Π is the perimeter of the cross section. This mass flow recovers Knudsen’s original expression for free molecular mass flow in circular channels^22,23^. Normalizing yields the nondimensional flow:

2 $$G=\dot{m}\frac{3\Pi L}{8{A}^{2}\Delta p}\sqrt{\frac{\pi RT}{2M}}.$$

Here, $\dot{m}$ is the mass flow, *L* is the length of the channel, Δ*p* is the pressure difference between the inlet and outlet, *R* is the gas constant, *T* is the temperature, and *M* is the molecular weight. The Knudsen number used for plotting is computed using the mean free path calculated from viscosity; see the Methods section for details. The mean free path calculated from viscosity is used to ensure comparability between the different data sets and the viscosity is an objective measure. This does not influence the results of the model, as explained in detail in the Supplementary Text 3. The actual mass flow decreases with larger Knudsen numbers. In the dimensionless representation, however, the typical phenomenon called the Knudsen minimum^22^ is clearly visible at approximately *Kn* = 1. This minimum is the result of the vanishing influence of convection and the rising contribution of diffusion at large Knudsen numbers.

The convective flow is derived from the Navier–Stokes equations with a modified version of the classical first-order slip approach^24^, and more details are given in the Methods section. In the literature, a slip coefficient is used to adjust the boundary condition to experimental data. This slip coefficient is then usually used to extract the tangential momentum accommodation coefficient (TMAC)^25^. The TMAC describes the ratio of diffuse to specular reflection of molecules at the surface ^26^. Although it has been shown that specular reflection cannot play a role in diffusion on technical surfaces and only starts to dominate when the roughness is very small^27^, fitting TMACs is widely used, and values typically range between 0.8 and 1^28^. Here, we show that it is not necessary to adjust the TMAC and therefore the slip coefficient for channels with circular cross sections. When comparing similar geometries with similar surface properties in terms of roughness and material, the TMAC should be equal. Therefore, the adjustment typically applied to the slip coefficient is more likely covering an effect arising from molecular diameters because the slip expression is dependent on the molecular diameter. Considering technical surfaces with TMAC equal to unity, the equation for slip flow through a circular cross section is:

3 $${\dot{m}}_{C}^{\text{circ}}={p}_{m}\Delta p\frac{\pi {D}^{4}}{128L}\frac{M}{\mu RT}\left(1+\frac{8Kn}{1+Kn}\right)$$

where *p*_*m*_ is the mean pressure between the inlet and outlet, *D* is the diameter of the circular channel and *Kn*(*d*) = λ(*d*)*/D*) is the mean Knudsen number in the channel. The influence of the molecular diameter *d* is caused by the mean free path λ(*d*); see Eq. (1).

Similar considerations yield an expression for rectangular channels based on an equation for slip flow by Jang et al.^29^, which is extended to account for the rising influence of the boundary for higher Knudsen numbers:

4 $${\dot{m}}_{c}^{rect}=\frac{w{h}^{3}{p}_{o}M\left(-CP1\right)}{32\mu RTL}\left[{\Pi }^{2}-1+2\frac{2-\sigma }{\sigma }\frac{Kn}{1+Kn}\frac{{p}_{m}}{{p}_{o}}\frac{CP2}{CP1}\left(\Pi -1\right)\right]$$

where *w* and *h* are the width and height of the channel, *p*_*o*_ is the outlet pressure, and σ is the TMAC. CP1 and CP2 are geometry-dependent coefficients with explicit formulations^29^. The characteristic length for calculating *Kn* is the channel height *h*.

The same dependency on the molecular diameter is given for the diffusive flow, which is modelled by a combination of Fickian self-diffusion ${\dot{m}}_{D,AA}$^30^ and free molecular flow ${\dot{m}}_{FM}$^14^. These two mass flows can be combined to yield an expression for the effective diffusive mass flow:

5 $${\dot{m}}_{D,eff}={\left(\frac{1}{{\dot{m}}_{D,AA}}+\frac{1}{{\dot{m}}_{FM}}\right)}^{-1}$$

In Eqs. (3) and (4), the Knudsen number is a function of the mean free path, as is the self-diffusion in Eq. (5). The mean free path, in turn, is a function of the molecular diameter, which explains the influence of the molecular diameter on the resulting mass flow:

6 $$\dot{m}={\dot{m}}_{C}+{\dot{m}}_{D,eff}.$$

A detailed derivation of the model and the explicit formulation of Eq. (5) are given in the “Methods” section.

**Figure 2:**
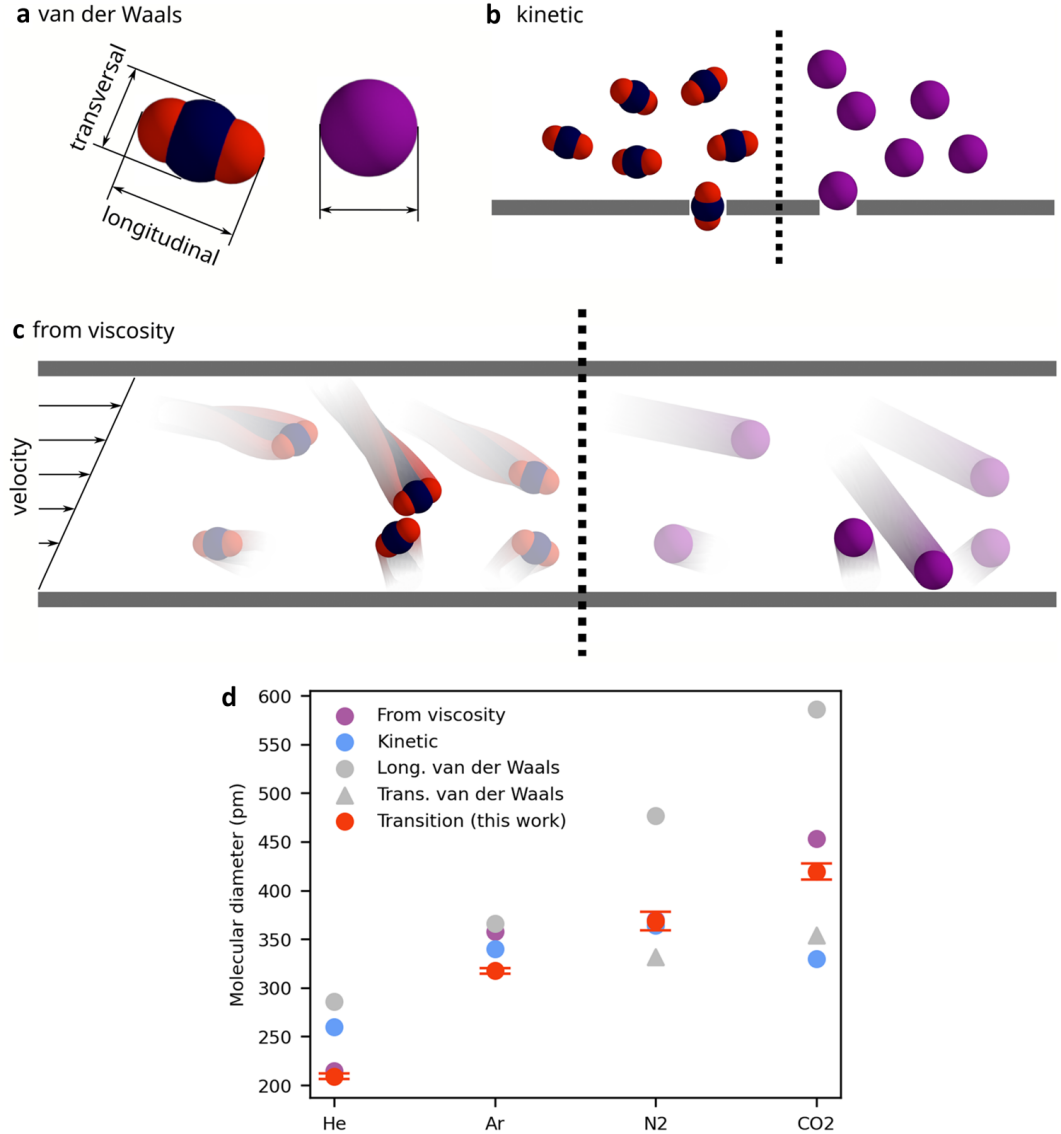
Compilation of molecular diameters for small gases. (**a**) The van der Waals diameter^5,6^ has two definitions for an asymmetric molecule like carbon dioxide while a spherical gas like argon just has one. (**b**) Molecular sieve experiments yield the kinetic diameter^4^. Molecular shape can have a large influence on the result. Here, carbon dioxide can pass the sieve in the right orientation, while argon is too large. This results in a larger kinetic diameter for argon than for carbon dioxide. (**c**) Viscosity (ν) can be measured by the momentum exchange from one moving boundary to a stationary one. The momentum exchanges through diffusion from fast moving molecules (top) to slow moving molecules (bottom). This diffusion is limited by the mean free path. While the two highlighted carbon dioxide molecules collide because of their size, the argon molecules can pass. The larger the molecules, the slower the molecules can diffuse, resulting in lower measured viscosity^9^. This results in a larger viscous diameter for carbon dioxide than for argon. (**d**) Actual molecular sizes. Error bars show the two-sided prediction interval of 95%. The kinetic diameter of nitrogen and the diameter calculated from viscosity are covered by the *transition diameter*. The two van der Waals diameters for nitrogen and carbon dioxide are the transversal and the longitudinal ones. For numerical values, see Supplementary Table S1.

Figure 2a–c gives an overview of the molecular diameters introduced before, namely, the kinetic diameter^4^, the van der Waals diameter^9,31^ and the diameter calculated from viscosity^9^. For the nonspherical molecules nitrogen and carbon dioxide, two different van der Waals diameters are given for the transversal and longitudinal orientations, respectively. Figure 2d shows a compilation of the different sizes of the diameters. The *transition diameter* introduced in this work by least squares fitting on experimental data of rarefied flow in microchannels is also included.

The mass flow according to Eq. (6) is calculated using these diameters for two geometries, which are the most investigated in the literature: rectangular and circular straight channels.

### Mass flow analysis

**Figure 3:**
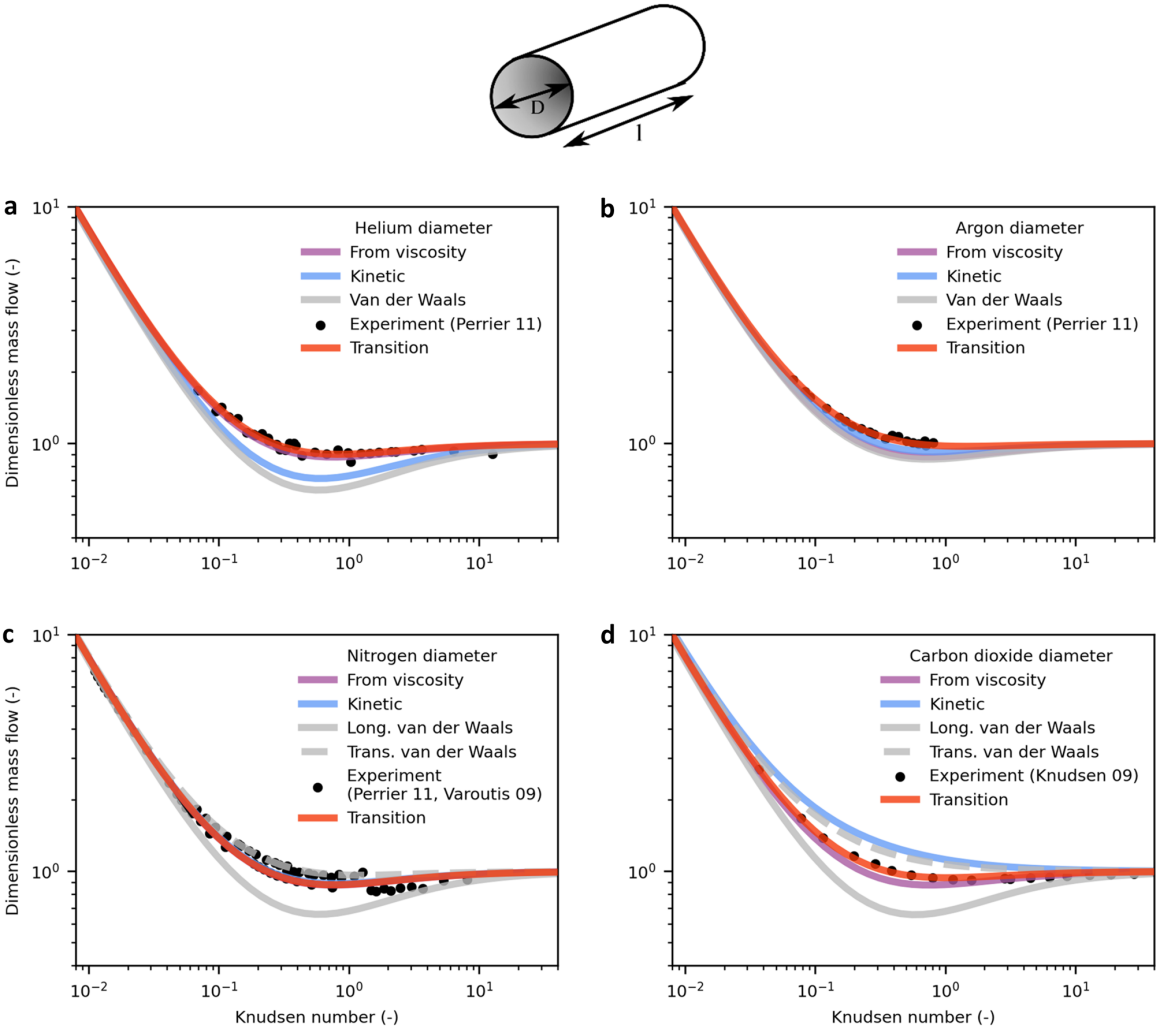
Mass flow in circular channels modelled with different molecular diameters compared to experimental data obtained from the literature. Dimensionless mass flow of helium^32^ (**a**), argon^32^ (**b**), nitrogen^32,33^ (**c**) and carbon dioxide^22^ (**d**) predicted by the model. The *transition diameter* is determined by least squares fitting to Eqs. (2)–(6). For comparison, the results for calculations using established molecular diameters from the literature are shown. For the nonspherical molecules nitrogen and carbon dioxide, two different van der Waals diameters are given for the transversal and longitudinal orientation, respectively.

Figure 3 shows the calculated mass flows together with the experimental results of gas flows through circular channels obtained from the literature for helium and argon^32^, nitrogen^32,33^ and carbon dioxide^22^.

For all diameters, the convective mass flow for *Kn* → 0 becomes identical, as in this range, the flow is dominated by the macroscopic property viscosity. Additionally, the free molecular mass flow for *Kn* → ∞ is equal because free molecular flow does not depend on molecular diameters but on the interaction with the geometry. However, different diameters show distinct mass flows at approximately *Kn* = 1 because here, the influence of slip is strong while the convective term is still present and self-diffusion is the dominating mechanism for diffusive flow. Both these terms are affected by the mean free path being in turn highly affected by the molecular diameter: the larger the molecule is, the smaller the mean free path. A smaller mean free path results in smaller diffusion and slip contributions and, therefore, in a smaller mass flow.

This influence of molecular diameters is demonstrated by the mass flows resulting from the van der Waals diameters (in case of nitrogen and carbon dioxide, the longitudinal diameters), which are larger than the others (see Fig. 2), leading to a clearly overpronounced minimum at *Kn* = 1. For argon, the deviation from data is less distinct. For the nonspherical molecules nitrogen and carbon dioxide, the smaller transversal diameter reproduces the data better than the larger longitudinal diameter.

The kinetic diameter is neither the largest nor the smallest diameter. It is close to the van der Waals diameter for helium and thus also predicts a very distinct minimum. In contrast, the kinetic diameter of carbon dioxide is close to the small transversal van der Waals diameter and overpredicts experimental data. For argon, the kinetic diameter yields a slightly lower mass flow than the data, while it results in a good prediction for nitrogen.

The diameter calculated from viscosity gives reasonable results for helium and nitrogen. The mass flow for argon and carbon dioxide is slightly underestimated. Of all the literature diameters, this diameter seems to be the most suitable one even though showing deviations to experimental data.

Because of the predictive nature of the model, it can be applied to experimental data of mass flows using the molecular diameter as a free parameter, yielding a new diameter. Since the model is most sensitive in the transitional regime, we use the expression *transition diameter.* A comparison between molecular diameters from the literature and the *transition diameters* is depicted in Fig. 2.

For the whole *Kn* range, the *transition diameter*-based model predicts the experimental mass flow data very well. Especially around the Knudsen minimum at *Kn* = 1, the transition diameter shows superior agreement with experimental data compared to the literature values of the established molecular diameters. For *Kn* → 0, reasonable agreement with experimental data was expected because the main transport mechanism, convection, is influenced mainly by viscosity which is constant over pressure. The viscosity is taken from the literature data where the values are obtained under continuum conditions, rendering it a suitable property for describing gas flow under these conditions. In addition, the mass flow for *Kn* → ∞ is convincingly predicted as a result of the dominating Smoluchowski expression in the free molecular regime, which is influenced only by the TMAC and the geometry of the channel and not by the molecular diameter, indicating that the TMAC is indeed unity for technical surfaces. A smaller TMAC would result in a higher mass flow for the free molecular regime, which is not represented by the experimental data. The good prediction for *Kn* = 1 is the result of the superposition of the different transport mechanisms at work. This agreement allows for the determination of a valid molecular diameter in this region.

**Figure 4:**
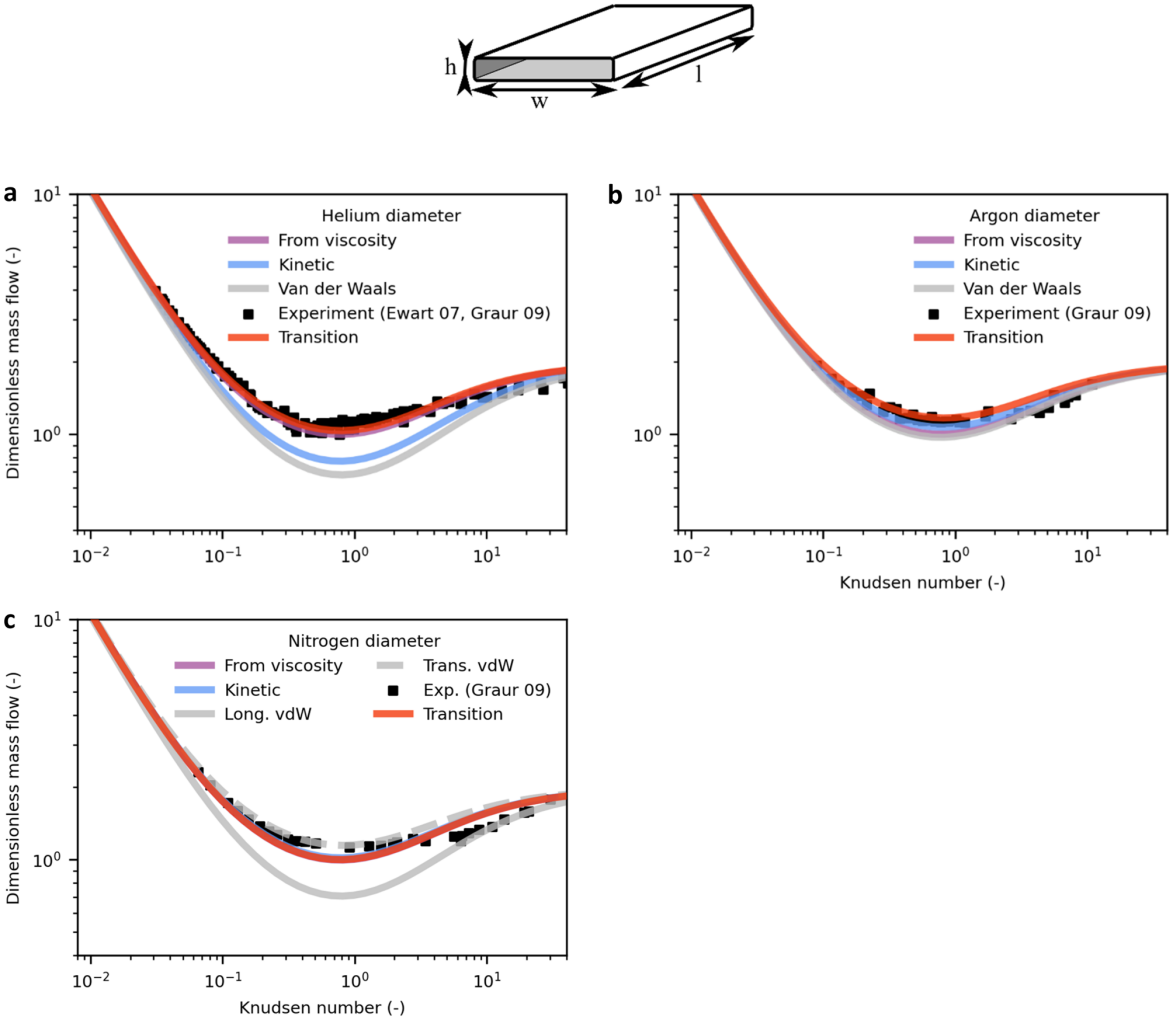
Mass flow in rectangular channels modelled with different molecular diameters compared to experimental data obtained from the literature. Dimensionless mass flow of helium (**a**), argon (**b**) and nitrogen (**c**) predicted by the model. The *transition diameters* are obtained from data on circular channels. For comparison, the results for different typical literature diameters are shown. For the nonspherical molecule nitrogen, two different van der Waals diameters are given for the transversal and longitudinal orientation, respectively.

Furthermore, the validity of the model can be confirmed by transferring it to rectangular cross sections, as shown in Fig. 4. Here, *transition diameter* values obtained before are used as well as data for helium^25,34^ and for argon and nitrogen^25^. Comparing literature data shows that the TMAC is found to be unity exclusively for circular cross sections, while the TMAC is 0.8–0.9 for rectangular cross section^28^, in agreement with our model, which provides good results when adjusting the TMAC to 0.9 for the slip expression. Since TMAC is defined as the ratio between diffuse and specular reflection, the values should be equal for surfaces with similar roughness made from a similar material. Apparently, the TMAC for the slip expression is also a function of cross-sectional shape, whose expression has not yet been established. To account for this cross-section effect, the TMAC in the slip expression is adjusted, while the TMAC for the free molecular flow is still unity. A clear indicator that the TMAC should in fact be unity for technical surfaces is the mass flow in the free molecular regime for *Kn* → ∞, as discussed above. See Supplementary Fig. 2 for details. For specular reflection, an extremely even surface is needed^27^. This contradiction could point to a flaw in the slip expression for rectangular channels.

The well-known Knudsen minimum at *Kn* = 1 is properly predicted by the model. The minimum is more pronounced for high-ratio rectangular channels than for circular channels, which is a phenomenon widely known in the literature^15^. The reason for this phenomenon is the geometry-dependent behaviour of free molecular flow as expressed by the Smoluchowski Equation: the flow through an infinitesimal cross-sectional element is proportional to the mean of its distances to equiangular spaced points on the boundary at the element’s axial position, since these distances are proportional to the distances molecules are free to travel. For rectangular channels, the sum of the mean distances of all cross-sectional elements is larger than the sum of the mean distances of a circular channel with equal hydraulic diameter and thus yields a larger nondimensional mass flow in the free molecular regime. This effect is the more enhanced the larger the channel’s aspect ratio becomes.

Although rectangular geometries are more intricate than circular geometries, the model represents the experimental data well with *transition diameters,* resulting in the best agreement with the data, demonstrating the predictive capability of the model with a known molecular diameter.

### Error analysis

**Figure 5:**
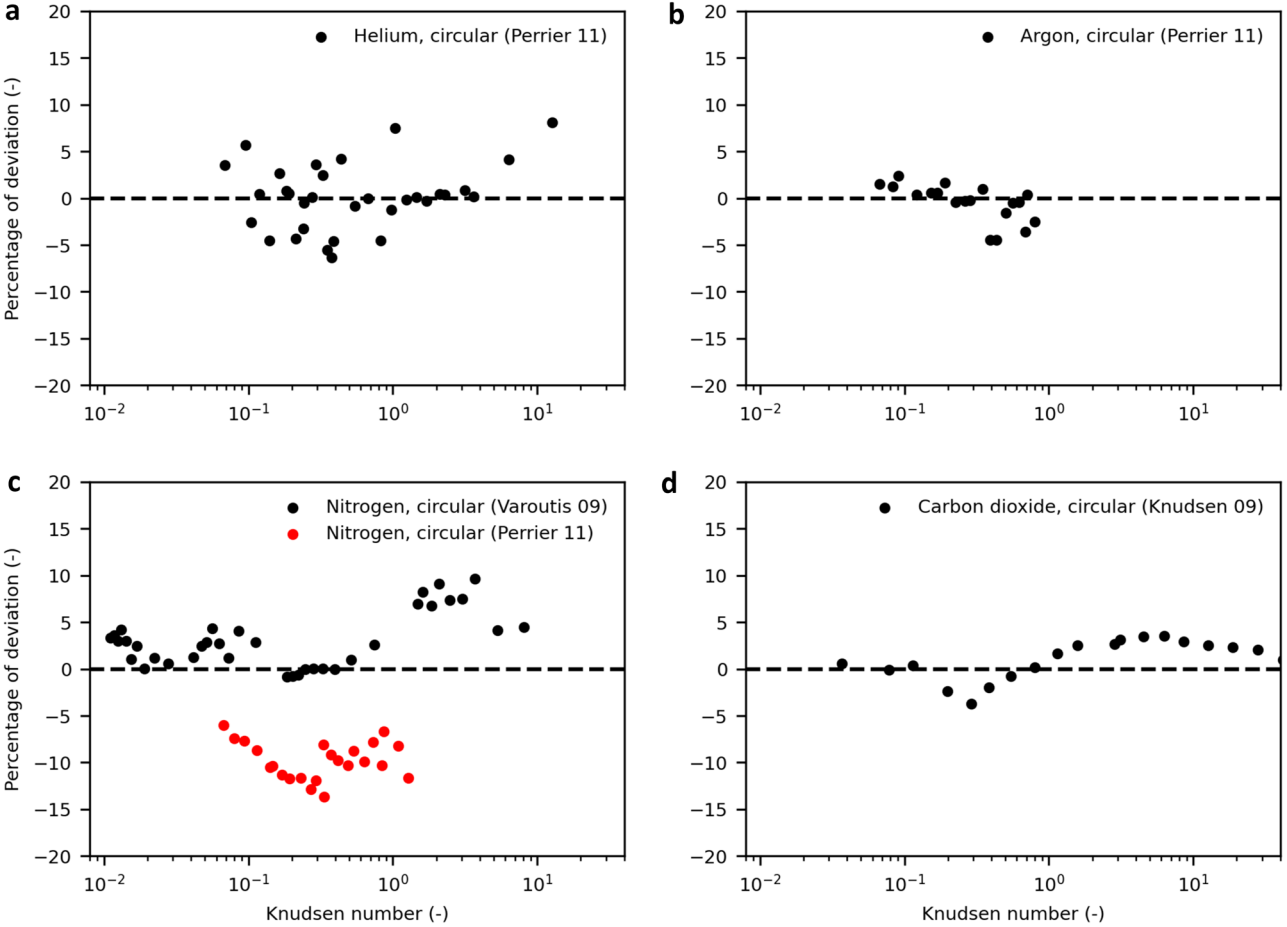
Deviation of modelled mass flow from experimental data for circular channels. The deviation is scattered without any discernible pattern, except for carbon dioxide, a strong indicator that the proposed model explains the experimental data well.

To analyse the quality of the model, the deviations between the modelled mass flow and experimental data are investigated. For a model explaining the data well, these deviations should occur only due to noise in the measurement data. This is the case for the circular channels, as seen in Fig. 5: the deviations are scattered around zero for all Knudsen numbers. Carbon dioxide is an exception, but those deviations are small. For nitrogen, the systematic error originating from two different sources becomes apparent.

**Figure 6:**
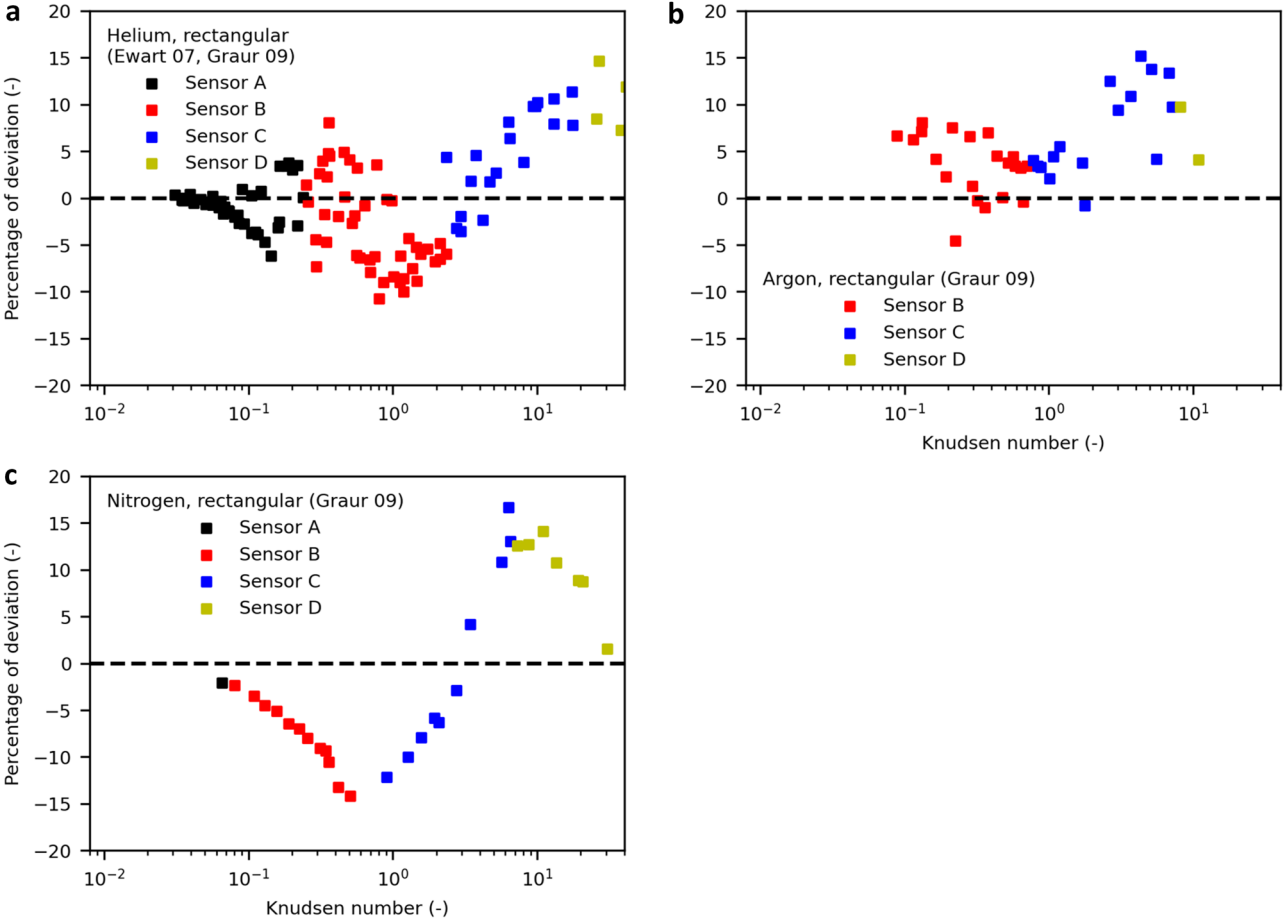
Deviation of modelled mass flow from experimental data for rectangular channels. The deviation shows some pattern that is strongly correlated to the pressure sensors used in different Kn regions.

The systematic deviations for the rectangular channels presumably result from measurement uncertainties of pressure sensors, as shown in Fig. 6. Although the deviations are scattered due to measurement noise, the distribution is not even around zero for all Knudsen numbers. For nitrogen and helium in regions of *Kn* < 3, the modelled mass flow is smaller than the experimental data. For regions of *Kn* > 3, the modelled mass flow is larger but then approaches the experimental data again. For argon, the same behaviour is observed, with an additional offset. A possible reason for this deviation pattern is the pressure regimes that are covered by different pressure sensors. Each pressure sensor exhibits a linear increase or decrease in deviation, so the systematic error of the model is likely due to measurement uncertainty, which is naturally largest at the outer region of each sensor measurement domain.

### Diameter analysis

An interesting observation on the order of the size of molecular diameters can be made from Fig. 2b. Apart from the van der Waals diameter consistently being the largest for each gas, the diameters show no order. The kinetic diameter is larger than the diameter calculated from viscosity for helium but smaller for nitrogen, argon and carbon dioxide. The *transition diameter,* however, is smaller than the diameter calculated from viscosity for helium, argon and carbon dioxide but only smaller than the kinetic diameter for helium and argon. The *transition diameter* is almost equal to the kinetic diameter and the diameter calculated from viscosity for nitrogen and larger than the kinetic diameter for carbon dioxide. The smallest deviation from the *transition diameter* shows the viscous diameter for helium, nitrogen and carbon dioxide and the kinetic diameter for argon. Bondi states that the van der Waals diameter changes less for heavy atoms^5^, which could explain why argon shows the smallest range of diameters.

There are different trends when comparing the same diameter across gases. The van der Waals diameters and the diameters calculated from viscosity increase from helium via argon and nitrogen up to carbon dioxide. The same applies to the *transition diameter*. The kinetic diameter, however, follows this trend up to nitrogen but is smaller for carbon dioxide than for nitrogen and argon. This can be explained by the measurement principle for kinetic diameters, which are obtained by molecular sieving experiments. Carbon dioxide can align lengthwise to pass very small pores, but this size is not representative of diffusion because it does not account for the longitudinal extent of the molecule.

These inconsistencies can be attributed to different molecules behaving very differently in certain situations. On the one hand, the shape of molecules, which may be nonspherical, can contribute to this. On the other hand, because of their nonrigid nature, the diameter can be interpreted as an apparent size that changes with environmental conditions and is not exclusively geometrical. These environmental conditions are dominated by intermolecular interactions for the viscous regime, which become less significant for higher Knudsen numbers, and by molecular-surface interactions for measurements of the kinetic diameter, which are less considerable for smaller Knudsen numbers. In the transitional regime of approximately *Kn* = 1, both of these effects in sum are minimal. The *transition diameter* is, as all the other singular diameters, an effective diameter describing the flow and diffusion behaviour and is therefore capable of describing non-spherical molecules as well.

## Discussion

To describe the behaviour of gases, the molecular diameter is a crucial parameter. The diameters found in the literature are either acquired under continuum conditions or under conditions that are dominated by gas-surface interactions. However, many applications, such as swing-adsorption processes, catalysis and space propulsion systems, operate at Knudsen numbers between 0.1 and 10. In these scenarios, the molecular diameter acquired at *Kn* → 0 or *Kn* → ∞ does not reflect the different surrounding conditions. The method presented here can determine a molecular diameter that is sensitive to the region around *Kn* = 1 and therefore represents a diameter that is valid in the transitional regime. This *transition diameter* is suitable to explain the behaviour of gas flow in this region, where the proposed model is most sensitive to the molecular diameter. This can be a valuable addition to the existing diameters, which are corresponding to conditions at *Kn* → 0 or *Kn* → ∞. For the continuum regime, using the viscosity is a reasonable approach. However, as soon as rarefaction effects become important, that is, for *Kn* > 0.01, using the *transition diameter* allows for describing gas flows more accurately. For the many relevant applications in the field of rarefied gases, we propose to use the presented method to extract the corresponding *transition diameter* and apply it to calculations for gases under rarefied conditions.

## Methods

The model for calculating the mass flow consists of convective and diffusive parts.

### Convection—circular channels

By assuming low Mach and Reynolds numbers, a gas can be treated as incompressible, and the mass flow through a circular channel can be expressed by the Hagen-Poiseuille law^35^:

7 $${\dot{m}}_{HP}={p}_{m}\Delta p\frac{\pi {D}^{4}}{128L}\frac{M}{\mu RT}.$$

The slip influence is modelled using the slip boundary condition proposed by Maxwell^24^:

8 $${u}_{s}={\left.-\alpha \lambda \frac{du}{dr}\right|}_{r=D\text{/}2}=-\alpha \frac{\lambda D}{4\mu }\frac{dp}{dx}=\alpha \frac{\lambda D}{4\mu }\frac{\Delta p}{L}$$

where *u*_*s*_ is the velocity difference between the wall and the gas directly at the wall. *du/dr* is the directional derivative of the gas velocity normal to the wall. λ is the mean free path of the gas and depends on the molecular diameter via Eq. (1), and α is expressed as:

9 $$\alpha =\frac{2-\sigma }{\sigma }$$

where σ is the tangential momentum accommodation coefficient TMAC, representing the fraction of molecules scattered diffusely to specularly reflected^24^.

The slip flow is the density-weighted slip velocity over the cross section:

10 $$\begin{aligned}{\dot{m}}_{S}= & \, \rho {u}_{s}\frac{\pi }{4}{D}^{2}\\ = & \, {p}_{m}\frac{M}{RT}{u}_{s}\frac{\pi }{4}{D}^{2}\\ = & \,{p}_{m}\Delta p\frac{\pi {D}^{3}}{16L}\frac{M}{\mu RT}\alpha \lambda \end{aligned}$$

The convective slip flow can be expressed by the addition of the Hagen-Poiseuille flow and the contribution of the slip flow:

11 $$\begin{aligned}{\dot{m}}_{c}= & \,{\dot{m}}_{HP}+{\dot{m}}_{S}\\ = & \,{p}_{m}\Delta p\frac{\pi {D}^{4}}{128L}\frac{M}{\mu RT}+{p}_{m}\Delta p\frac{\pi {D}^{3}}{16L}\frac{M}{\mu RT}\alpha \lambda \\ = & \,{\dot{m}}_{HP}\left(1+8\alpha \frac{\lambda }{D}\right)\end{aligned}$$

For higher Knudsen numbers, the wall influence begins to influence the mean free path. This was stated by Bosanquet, as described by Pollard and Present^36^: each step of the random walk that defines the diffusion process is terminated either by an intermolecular collision or by the surrounding geometry. These collision frequencies can be simply added. Since the collision frequencies are inverse to the mean free path, this results in an effective mean free path:

12 $${\lambda }_{eff}={\left(\frac{1}{\lambda }+\frac{1}{{L}_{c}}\right)}^{-1}$$

Using this effective mean free path in Eq. (11), the result for the convective flow is:

13 $$\begin{aligned}{\dot{m}}_{c}= & \,{\dot{m}}_{HP}\left[1+8\alpha \frac{{\left(\frac{1}{\lambda }+\frac{1}{{L}_{c}}\right)}^{-1}}{{L}_{c}}\right]\\ = & \,{\dot{m}}_{HP}\left[1+8\alpha {\left(\frac{1}{Kn}+1\right)}^{-1}\right]\\= & \,{\dot{m}}_{HP}\left[1+8\alpha \frac{Kn}{1+Kn}\right]\end{aligned}$$

Because λ depends on the molecular diameter, see Eq. (1), so does λ_*eff*_ and therefore the slip flow in Eq. (13), as long as *Kn* is sufficiently small so that the characteristic length *L*_*c*_ of the geometry does not predominate the mean free path λ. Combining Eqs. (13) and (7) and setting α to unity, as explained in the discussion section, yields Eq. (3), which is the convective part of the model for determining the molecular diameters.

### Convection—rectangular channels

To cover all aspect ratios of rectangular channels, a modification of an expression by Jang et al.^29^ is used. *Kn*_*o*_ from the original expression is replaced by $\frac{{\lambda }_{eff}}{h}\frac{{p}_{m}}{{p}_{o}}=\frac{Kn}{1+Kn}\frac{{p}_{m}}{{p}_{o}}$ to account for the wall influence by using the effective mean free path from Eq. (12) with *L*_*c*_ = *h*, yielding Eq. (4). For a quadratic cross-section, the geometry-dependent coefficients, which can be calculated analytically, result in *CP1* = − 0.5623 and *CP2* = − 4.4985, while for an aspect ratio of 0.02, *CP1* = − 1.3165 and *CP2* = − 7.9189.

### Diffusion

The diffusive flow is modelled by a combination of Fickian self-diffusion^35^ and free molecular flow. The self-diffusion results from Fick’s first law^37^:

14 $${J}_{AA}={D}_{AA}\frac{\Delta c}{L}$$

where *J*_*AA*_ is the molar flux, *D*_*AA*_ is the self-diffusion coefficient, and Δ*c* = *c*_*i*_ − *c*_*o*_ is the concentration difference between the inlet and outlet, which can be expressed in terms of pressure as:

15 $$\Delta c=\Delta p\frac{1}{RT}.$$

The self-diffusion coefficient^30^ is calculated by:

16 $${D}_{AA}=\frac{1}{3}\lambda v$$

where *v* is the mean molecular velocity

17 $$v=\sqrt{\frac{8RT}{\pi M}}.$$

The corresponding mass flow then is:

18 $${\dot{m}}_{D,AA}=MA{J}_{AA}=\Delta p\frac{1}{3}\lambda v\frac{A}{L}\frac{M}{RT}.$$

However, for large Knudsen numbers, the influence of the surrounding geometry begins to dominate the flow, which is well described using the free molecular flow expression by Smoluchowski^14^ [Eq. (6) and expression with α (TMAC) on p. 1567]

19 $${\dot{m}}_{FM}=\alpha \frac{1}{2\sqrt{2\pi }}\sqrt{\frac{M}{RT}} \frac{\Delta p}{L}\Lambda$$

where Λ is a geometry dependent expression. For circular channels, this is:

20 $${\Lambda }^{\circ }=\frac{2{D}^{3}\pi }{3}$$

which recovers Knudsen’s original formula. For rectangular channels, the expression is:

21 $$\begin{aligned}{\Lambda }^{rect}= & \, 2\left[{h}^{2}wln\left(\frac{w}{h}+\sqrt{1+{\left(\frac{w}{h}\right)}^{2}}\right)\right.\\ + & \, h{w}^{2}ln\left(\frac{h}{w}+\sqrt{1+{\left(\frac{h}{w}\right)}^{2}}\right)\\ & \, \times \left.\frac{-{\left({h}^{2}+{w}^{2}\right)}^{3\text{/}2}}{3}+\frac{{h}^{3}{w}^{3}}{3}\right].\end{aligned}$$

Λ can also be numerically solved for arbitrary cross-sections. For more details, see the provided Python script.

To combine the two mechanisms of self-diffusive and free molecular flow, we introduce an approach based the reasoning of Bosanquet which is described in the derivation of the effective mean free path above. This is reasonable because the diffusion coefficient is proportional to the mean free path^30^ and the diffusive mass flows are proportional to the diffusion coefficients^35^. This yields an expression for the effective diffusive mass flow, see Eq. (5).

### Resulting mass flow and nondimensional form

The actual mass flow is just the addition of convective and diffusive flow; see Eq. (6). For plotting and fitting, the nondimensional mass flow calculated by Eq. (2) is used. The Knudsen number for plotting is calculated using the constant viscosity of the gas instead of its diameter to ensure uniformity, implying that the experimental data shown are independent of the molecular diameter.

22 $${Kn}_{visc}=\frac{{\lambda }_{m,visc}}{{L}_{c}}$$

Here, *L*_*c*_ is the characteristic length of the channel (the diameter in case of a circular channel, the height in case of a rectangular channel), and λ_*m,visc*_ is the mean free path calculated by:

23 $${\lambda }_{m,visc}=\frac{\mu }{{p}_{m}}\sqrt{\frac{\pi RT}{2M}}$$

where *p*_*m* _= (*p*_*in* _+ *p*_*out*_) / 2 is the mean pressure, and μ is the dynamic viscosity.

### Fitting procedure and error analysis

For determination of the *transition diameter*, the free parameter of the model is fitted to the experimental data by least square optimization. The data are used in their nondimensional form to avoid overweighting of high absolute mass flows for small Knudsen numbers. Theoretically, it would be sufficient to have data points just at *Kn* = 1 for determining the molecular diameter. However, it is important to be sure that the TMAC is set correctly, which can be validated by measurements in the free molecular regime. Additionally, the more data points are available, the smaller the uncertainty becomes. The prediction interval shown in Fig. 2 is calculated by:

24 $$d\pm {t}_{\alpha }s\sqrt{1+1/N}$$

where *s* and *N* are the variance and number of data points, respectively. *t*_α_ is the percentile of Student’s *t*-distribution corresponding to a confidence level of α^38^. In this case, α = 0.05 is used for a two-sided 95% confidence interval. The degree of freedom is *N *– 1 when fitting one parameter, the diameter in this case.

## Supplementary Information

Supplementary Information.

## Data Availability

The data that support the findings of this study are openly available in zenodo repository at https://zenodo.org/record/5902778.
