# Supplementary material for: Molecular diameters of rarefied gases
Source: Sci Rep. 2022 Feb 8;12:2057. doi: 10.1038/s41598-022-05871-y (PMC8825840; doi:10.1038/s41598-022-05871-y)
Supplement: Supplementary file 1 — Supplementary Information. [file 41598_2022_5871_MOESM1_ESM.docx]

**Supplementary Materials**

**Supplementary Text 1**

**Calculation of Knudsen numbers for Fig. 1A**

Adsorption materials: Pore sizes of 200 µm down to 0.1 nm. Large changes in pressure or temperature in swing-adsorption applications result in Knudsen numbers ranging from ~0 to 10.

Catalyst: Pore diameter distribution: 12 – 80 nm. Mean free path of air at 1 atm: 70 nm. This results in Knudsen numbers of 0.9 to 6.

Space propulsion systems: Typical length scales of 1 – 10 cm. Due to the big pressure differences between ignition chamber and free space, the Knudsen numbers range from 0.1 up to 100.

**Supplementary Text 2**

**Parameters of sample case for Fig. 1B**

For both plots A and B, a rectangular channel with h = 9.38 µm and w = 492 µm was used. The mass flow in plot A is the superposition mass flow of plot B. The mass flows in plot B where calculated using the respective parts of the model, that is Eq. (4) for the convective mass flow and Eq. (5) for the diffusive mass flow. The superposition mass flow is just the addition of convective and diffusive mass flow. For the shaded areas depicting the influence of the molecular diameter on the mass flow, the previous calculation is repeated with different molecular diameters. For the actual mass flow plot, a diameter of 200 pm was used. For the upper and lower bound of the shaded area, 160 pm and 250 pm where used, corresponding to a 25 % decrease and increase, respectively.

**Supplementary Text 3**

**Details on why the mean free path calculated from viscosity is used for the Knudsen number for plotting**

To compare two systems in a non-dimensional form, the de-dimensionalization must be applied in the same way for both systems. In this case, one system would be the experimental mass flows, the other system would be the analytical model with one of the diameters. The two important variables for comparison are the pressure of the system and the mass flow itself. The mass flow is uniformly de-dimensionalized using eq. (2), where no ambuigity arises. To de-dimensionalize the pressure, the Knudsen number is used. However, the Knudsen number contains the mean free path and is therefore dependent on the molecular diameter.

To compare the experimental data with the model, the same de-dimensionalization of the pressure must be used, therefore the same Knudsen number and the same molecular diameter for calculating the mean free path is needed. Using the respective diameter for de-dimensionalization of each model version, for a meaningful comparison the experimental mass flows would need to use that diameter as well for de-dimensionalization. This would result in a mere x-axis shift, as shown exemplary in the Supplementary Figure S1 included below. This x-axis shift would apply to both the model and the experimental data in the same way. Also, to avoid a cluttered plot, one would need to create a separate figure for each diameter, because all experimental data points would have slightly different positions for each diameter. To summarize, we decided to use the viscous diameter as an objective quantity to uniformly de-dimensionalize all experimental data and model results. Doing so differently would not change the determined sizes for a transition diameter.

**Supplementary Text 4**

**Calculation of van der Waals diameters for nitrogen and carbon dioxide**

Because of the non-spherical structure of nitrogen and carbon dioxide, the van der Waals diameters used in this manuscript are derived from the van der Waals diameters found in literature for the elements as well as the binding length. For nitrogen, the transversal diameter is just the diameter of a nitrogen atom^1^. The longitudinal diameter is the diameter of one nitrogen atom plus the bond length between to nitrogen atoms which is 145 pm^2^ since the bond length is defined from center to center. For carbon dioxide, the transversal diameter is just the diameter of a carbon atom, which is bigger than the oxygen atom. The longitudinal diameter is calculated by *r_c_ + bond(CO) + bond(OC) + r_c_* which is equal to the diameter of a carbon atom plus two times the bond length between a carbon and an oxygen atom which is 116 pm^2^.

**Supplementary Text 5**

**Data preparation**

For some experimental data from literature, no raw data is available. In these cases the data was extracted graphically from the plots using a software called WebPlotDigitizer (https://automeris.io/WebPlotDigitizer) which is a very precise method because non-distorted images directly from the online versions of the papers were used. These plots often show a non-dimensional mass flow over the Knudsen number or something similar. To get the raw experimental data from this, the pressure ratio *p_r_* is used to acquire the inlet and outlet pressures via

|  | $p_{o}=\frac{2p_{m}}{p_{r}+1},p_{i}=p_{o}p_{r}.$ | (1) |
| --- | --- | --- |

The mean pressure *p_m_* in turn can be calculated from *Kn*. Fortunately, neither the plots because of the form of the dimensionless mass flow nor the method itself for determining molecular diameters is sensitive to the actual pressure ratio. Therefore, in the cases where assumptions had to be made, these assumptions are irrelevant to the result. Data of Knudsen was truncated to suppress an over-weighted influence of the viscous regime. For an overview, see Table S2.

**Supplementary Figure 1**

| 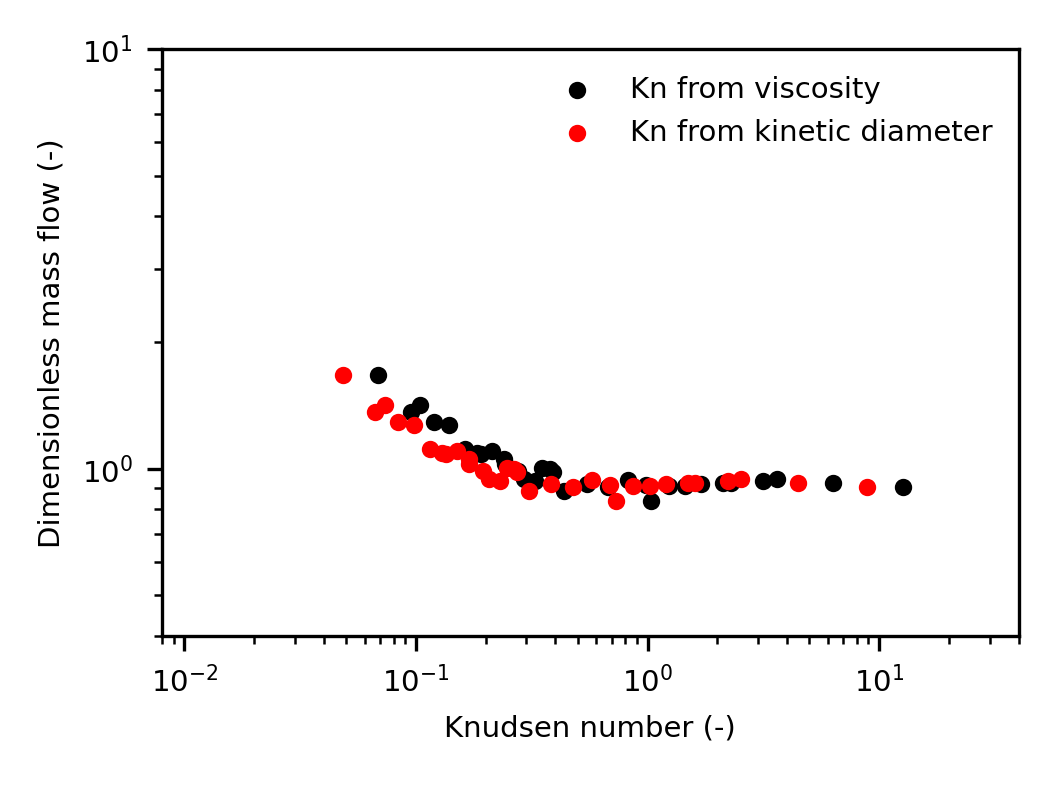 |
| --- |
| Fig. S1 Dimensionless mass flow of helium in a circular tube^4^. Knudsen number calculated in two different ways. |

**Supplementary Figure 2**

| 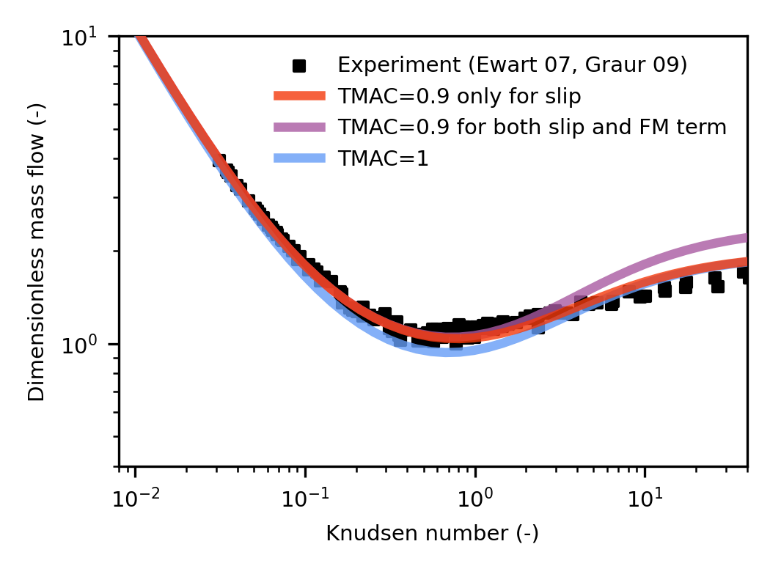 |
| --- |
| Fig. S2 Dimensionless mass flow of helium in a rectangular channel, calculated with the transition diameter but different TMACs for the slip convection term and the free molecular term |

**Supplementary Table 1**

| **Table S1 Molecular diameters in pm** | | | | |
| --- | --- | --- | --- | --- |
| Gas | *Transition*  (present work) | From viscosity^29^ | Kinetic^34^ | Van der Waals^1,29,30^ |
| Helium | 209 +- 3 | 215 | 260 | 286 |
| Nitrogen | 369 +- 9 | 370 | 364 | 477, 332 |
| Argon | 317 +- 3 | 358 | 340 | 366 |
| Carbon dioxide | 419 +- 8 | 453 | 330 | 586, 354 |

**Supplementary Table 2**

| **Table S2 Details on experimental data from literature** | | |
| --- | --- | --- |
| Data source | Assumptions and truncation | Material and geometry |
| Perrier 11 ^4^  (He, N2, Ar circ.) | None (from plot) | Silica; *D* = 49.6 µm; *L* = 1.82 cm |
| Ewart 07 ^5^  (He, rect.) | None (raw data) | Silicon; *h* = 9.38 µm;  *w* = 492 µm; *L* = 9.39 mm |
| Graur 09 ^6^  (He, N2, Ar rect.) | None (from plot) | Silicon; h = 9.38 µm;  *w* = 492 µm; *L* = 9.39 mm |
| Varoutis 09 ^7^  (N2, circ.) | None (raw data) | Stainless steel; *D* = 16 mm;  *L* = 1.277 m |
| Knudsen 1909 ^821^  – Roehre Nr. 4  (CO2, circ.) | Raw data. Truncation of the first 8 data points of the viscous regime to focus the fit on the transitional regime.  No pressure ratio given; assumption of a ratio of 5 | Glass; *D* = 6.66 mm; *L* = 2 cm |

1. Alvarez, S. A cartography of the van der Waals territories. *Dalt. Trans.* **42**, 8617 (2013).

2. Lide, D. R. *CRC handbook of chemistry and physics: a ready-reference book of chemical and physical data*. (CRC Press, 1995).

3. Baker, R. W. *Membrane Technology and Applications*. (John Wiley and Sons Ltd, 2012). doi:10.1002/9781118359686.ch2.

4. Perrier, P., Graur, I. A., Ewart, T. & Méolans, J. G. Mass flow rate measurements in microtubes: From hydrodynamic to near free molecular regime. *Phys. Fluids* **23**, 42004 (2011).

5. Ewart, T., Perrier, P., Graur, I. A. & Méolans, J. G. Mass flow rate measurements in a microchannel, from hydrodynamic to near free molecular regimes. *J. Fluid Mech.* **584**, 337 (2007).

6. Graur, I. A., Perrier, P., Ghozlani, W. & Méolans, J. G. Measurements of tangential momentum accommodation coefficient for various gases in plane microchannel. *Phys. Fluids* **21**, 102004 (2009).

7. Varoutis, S., Naris, S., Hauer, V., Day, C. & Valougeorgis, D. Computational and experimental study of gas flows through long channels of various cross sections in the whole range of the Knudsen number. *J. Vac. Sci. Technol. A Vacuum, Surfaces, Film.* **27**, 89–100 (2009).

8. Knudsen, M. Die Gesetze der Molekularströmung und der inneren Reibungsströmung der Gase durch Röhren. *Ann. Phys.* **333**, 75–130 (1909).
